# The Instruction of Patients in the Personal Care of the Mouth

**Published:** 1921-07

**Authors:** Justin D. Towner


### THE INSTRUCTION OF PATIENTS IN THE PERSONAL CARE OF THE MOUTH

BY JUSTIN D. TOWNER, D.D.S.

Accepting the meaning of hygiene as the "science of preserving health," and prophylaxis as that of "preventing disease," the two seem synonymous at first thought. True, in the preservation of health, disease is prevented, but the procedures by which each is accomplished are sufficiently varied to leave them synonymous mainly in the fact that each has as its object the preservation of health in a specific form.

Dental hygiene relates to the removal of predisposing conditions and is accomplished by sanitation. In this respect it is distinctly a defensive measure and as such is chiefly the obligation which the patient must assume.

Prophylaxis refers to the destruction of exciting causes and is accomplished by disinfection. This being true, it is an aggressive procedure and is, therefore, mainly the obligation of the dentist.

It is evident then that the responsibility must be at least equally divided between patient and dentist, and that sole reliance can no longer be placed in either hygiene or prophylaxis as a means of maintaining a healthy mouth. The sooner a dentist admits the mistake of assuming a greater obligation than possible to discharge, the more apt he will be to demand the thorough co-operation of the patient in all matters of mouth hygiene.

Before expecting intelligent co-operation on the part of the patient, the dentist must wholly accept the responsibility and discharge the obligation of applied instructions. The word applied is used advisedly to convey the idea that all procedures advised for the preservation of oral health should be actually demonstrated in the patient's mouth. All efforts made to educate patients up to a proper appreciation of healthy mouths, and the responsibility which they must necessarily assume in this respect should be coincident with any and all other services rendered.

### USE OF TEETH

The first, and perhaps the most important, instruction to be given is that concerning the proper use of teeth. In this age of strenuosity and super-prepared foods people seem to have forgotten the original function of the dental apparatus. Even dentists, who more than all others should know this and its relative value in preserving the health of these organs, are apt to overlook the importance of the use of teeth and to concentrate every effort upon their care. Any instructions, then, which do not stress the proper use of teeth are not sufficiently complete, to say the least.

The real purpose of teeth should be explained and the movements of the mandible necessary to the act of chewing should be demonstrated. The beneficial influences of correct mastication upon the health of teeth and their investing tissues, as well as upon digestion and nutrition, should be impressed upon patients. To more immediately overcome any apparent evil, resulting from lack of use, the patient may be advised to chew gum for ten or fifteen minutes after meals, or at least three times a day. The bolus should be large enough to insure its cleansing and massaging effect, and sufficiently tough to require considerable masticatory energy. The stimulation of the salivary glands, resulting in increased flow of saliva, is beneficial both to the dental and digestive organs, and this alone is sufficiently advantageous to justify its endorsement, even though other benefits such as exercise, cleaning, massaging and inducing the habit of mastication were not so obvious.

### DIET

Diet does not necessarily mean a restriction in the selection of foods. With the exception of well-established organic deficiencies demanding reduction, or complete elimination of certain food elements, the question is only suggestive of a properly balanced ration. The selection, combination and proportion of food with respect to age, climate and occupation is necessary to normal nutrition, the first of the three fundamental laws of life. Exercise and breathing are the other two laws, and the simple rules governing each should be thoroughly impressed upon patients as necessary to health. If dependence upon natural processes is admitted in the prevention and cure of disease, then these simple fundamental laws of health should be well understood.

The selection, preparation, mastication and insalivation of food is most essential, yet it is that which is usually given least consideration. One who makes a mere trash-hopper of the mouth must not expect it to be kept clean, or to enjoy the blessings of good health. The exercise of common sense in the matter of diet is the greatest defense against disease in any part of the body.

### TONGUE SCRAPER

There is probably no part of the mouth less often cleaned than the tongue, which, in many cases, is a veritable hotbed of infection continually contaminating one's food, drink and breath. This is certainly detrimental to mouth cleanliness, to say the least, and, for such reason alone, should receive adequate attention. A valuable mouth toilet requisite, especially designed for cleaning this part of the oral cavity, is the tongue scraper.

The simplest of the several patterns obtainable should be selected and instructions given as to its use and care, particular emphasis being placed upon its sterilization.

The tongue should first be protruded as far as possible directly from the mouth and curved slightly toward the chin. While in this position the top, or palate surface, may be cleaned from dorsum to tip by means of a quick scraping motion. Next protrude the tongue laterally first to one side and then the other and clean the edges. The scraper should always be used at night before retiring, preferably night and morning, and ought to be followed by the brush.

The tendency to gag may be overcome by holding the tongue in its protruded position and inhaling while cleaning.

### SELECTION, USE AND CARE OF THE BRUSH

Of all mouth toilet requisites the brush is by far the most necessary. The benefits derived from its employment depend upon a correct understanding of its true office, careful selection, intelligent use and proper care.

If the term "mouth brush" could be generally applied to what is now known as the "tooth brush," a clearer understanding of its real purpose would naturally result. The name now applied suggests a too limited use to encourage its proper employment.

Examination should be made of each mouth to determine the brush best suited to the available space in different positions, and any peculiar conditions which may be present. The style of brush should be selected, and its use demonstrated in the mouth of the patient, by the dentist. This is not only a great service to the patient, but a real obligation, the discharge of which will be appreciated by those who are really interested in mouth cleanliness.

Brushes designed in accordance with the recommendations of the American Academy of Periodontology will prove most efficient, though these specifications may be slightly varied to suit unusual cases or individual preferences. The report of the council is an attempt at standardization which merits the approval of all who appreciate the effort to avoid poorly designed brushes and create opinion favorable to more definite and efficient forms.

While the brush form has much to do with its efficiency, the method of use is of greatest importance. It should be first used upon the teeth with a combination vibratory and pick motion. This consists of placing the bristle tufts upon the teeth and slightly moving the brush head in line with its long axis until the bristles are well seated in the embrasures, or sulci, etc., depending upon what surfaces are being cleansed, and then using the pick stroke. In this way the bristles reach the most inaccessible places, and not only dislodge and remove accumulations, but tend to polish those surfaces. This method permits the use of a smaller brush head than ordinarily indicated, thereby reducing the number of teeth to be cleaned at one operation and increasing efficient operation.

In addition to definite brush movements, certain positions and surfaces should be cleaned in the order of their hygienic importance. To simplify and make more effective the process of cleaning the dental apparatus, it is suggested that the arch be divided into molar, bicuspid and anterior divisions on each side of the median line, upper and lower, and that tooth surfaces be designated as embrasure, buccal, lingual and occlusal. By beginning with the upper right molar region and brushing the surfaces of each division in the manner and order named before passing to the next, the procedure is made more accurate. This process should follow in regular order from right to left in the upper arch and from left to right in the lower.

Often the buccal surfaces of the upper molars, especially the second and third, give evidence of not having been brushed. The reason for this, in the mouths of those who really make an honest effort, is the fact that the teeth are either closed tightly, thus constricting the masseter muscle, which in this position shields these surfaces from approach; or, if the teeth are held too far apart, this muscle is pulled down over these same surfaces and prevents the brush from reaching them. To gain access to and properly brush these areas the teeth should be slightly apart so that the masseter muscle will be relaxed.

The lingual surfaces of the lower molars also frequently escape attention either on account of the resistance the tongue offers to the intrusion of the brush, or to their lingual inclination. Both of these conditions may be present at the same time and make the cleaning of such surfaces exceedingly difficult. If the tongue is retruded and the brush introduced low enough, little trouble will be experienced in reaching and thoroughly brushing these areas.

After cleansing the teeth, and the bristles have become somewhat softened, the gums should be brushed with a sweeping motion from the crest toward the deflection. Following this the soft mucous tissues, including the tongue, should be swept in order to cleanse and stimulate them. A larger brush may be used upon the soft tissues than upon the teeth, though it is not by any means preferable.

In healthy mouths it is permissible to use a stiff brush, but in case of diseased and tender soft tissues the bristles should be softened to avoid injury to these structures.

After use the brush should be washed well and dipped in a solution of benzoic acid and sodium chlorid, or sprinkled with sodium chlorid and allowed to dry before being put into service again. The mouth wash, suggested later, is an excellent disinfectant for the brush. It is essential that the brush be kept as aseptic as possible to prevent the reinoculation of the soft tissues with pathogenic organisms.

### SELECTION AND USE OF FLOSS TAPE

The proper selection and intelligent use of floss tape is essential to the maintenance of mouth hygiene. Care must be exercised in selecting that width which will easily pass between all contact points, because when much force is used to get the floss to place it is apt to snap past these points and injure the gingivae. If Salter's tape is used, the Nos. 2 or 3 will prove most desirable in the majority of cases. Occasionally, however, No. 1, the narrowest, or No. 4, the widest, will be suggested by the general conditions presented.

The correct use of floss is a procedure which can seldom be made clear by description alone. Its use must be demonstrated in the mouth and this supplemented by having the patient use the tape on all teeth, under supervision. The avoidance of either plunging the tape on to the soft tissues or passing it so far underneath the gingivae that the peridental membrane attachment will be injured should always be advised. A violent sawing motion must not be adopted, and its withdrawal should be buccally or lingually in order to more definitely remove debris.

It should be seen that all contact points will permit the passage of tape; and in cases of fixed bridgework, involving two or more abutments with dummies touching the ridge, a bodkin made of small brass ligature wire, looped and twisted at the end, may be furnished for threading the tape to place. If the tape is loaded with a non-abrasive paste or powder it will clean better.

The importance of cleansing the teeth with tape is proven by the fact that there are sixty-four surfaces which the brush can not reach, and that these are more subject to disease.

### SELECTION AND USE OF PICKS

Before middle life tooth picks are seldom necessary to mouth hygiene. Beyond this age the septal tissues are often found receded to such an extent that with discrimination in selection and care in use the pick may prove a valuable prophylactic requisite. They should be thin, flat and narrow, and made of either close fibered wood, highly polished, or of quill.

Picks are primarily intended to clean the approximal surfaces of teeth between the crest of the gingivae and the contact point, but may also be employed to good advantage on the exposed labial and buccal areas. They should be used with a rubbing motion direct from the gingival margin toward the incisal or occlusal angles.

Care must always be exercised to avoid unnecessary injury and forced recession of the septal gingivae. The habit of forcing picks between the approximal surfaces of teeth and continually prodding the septal tissues is pernicious, therefore patients should never use picks except upon the advice of, and after careful instruction, by the dentist.

SELECTION AND USE OF DENTIFRICE

There can be no doubt but that the value of dentifrices has been greatly exaggerated. This is evidenced by the great number of these preparations now upon the market and the extravagant claims made for many of them. There seems not so much thought given to the fact that dentifrices are mere aids in mechanical cleaning as is devoted to the compounding of highly medicated and extravagantly flavored preparations. Most dentifrices are alkaline, as they should be, but unfortunately many of these are salivary depressants, which is directly opposed to health except in rare cases of excessive secretion. There can be no objection to having a preparation more strongly alkaline than saliva, provided it contains an ingredient which will at least counteract its otherwise depressant effect.

A dentifrice, then, should be composed of agents which will clean without abrading, promote activity of the salivary glands, neutralize acidity and be antiseptic. A simple and efficient preparation of this nature is composed of sodium chlorid, sodium perborate, benzoic acid, with floated calcium phosphate or cellulose base. While it is not within the province of this discussion to treat of the merits of these particular ingredients, or the combination as a whole, it will require little thought to determine that such a preparation meets the demands of a safe and sane dentifrice.

The powdered form is more efficacious, especially when used dry, as it not only cleanses, but polishes tooth structure. Such a preparation should be used twice daily, preferably upon rising in the morning and just before retiring at night. If, for any reason, once a day is deemed sufficient, then this should be at night when the most complete mouth toilet is necessary.

### SELECTION AND USE OF MOUTH WASH

A mouth wash is generally indicated in diseased conditions, and when judiciously selected and used is a potent factor in stimulating the tissues to rapid recovery of normal function. Even in a healthy mouth a well-balanced wash, properly used, will tend to reduce acid forming organisms without exerting any paralyzing effect upon tissue. In fact, a wash composed of isotonic sodium chlorid, as a base, with an aqueous solution of benzoic acid and sodium borate in water-soluble menthol, will stimulate the flow of saliva, the normal mouth antiseptic, tend to inhibit pathogenic organisms, and induce the better care of oral tissues. It is not to be understood that the suggestion of an antiseptic wash is an advocacy of highly alcoholic or astringent preparations, either in disease or health; they have no place in a dental toilet armamentarium.

The manner of using a mouth wash should always be explained and demonstrated, as few know how best to employ it. Such a preparation should be used first as a gargle, then as a douche, to be forced between and around the teeth and finally on the brush, about one minute being consumed for each mouthful, or one fluid ounce.

Gargling is a difficult procedure for some and the dentist should make sure that those for whom it is prescribed can do it. As a substitute for gargling the spray, by means of an atomizer, may be used.

In forcing the solution between and around teeth and bathing the mucous membranes, considerable pressure by the muscles of the pharynx, cheek, lips and tongue must be exerted.

A bland wash comprising the agents mentioned may be employed several times daily without injury to either teeth or soft tissues, and is an excellent solution with which to sterilize the brush.

### FREQUENT VISITS

The necessity for frequent visits to the dentist should be explained to each patient. The importance and frequency of these visits depend, of course, upon one's susceptibility to mouth disease and ability to properly perform its toilet. Few people can dispense with the services of a dentist long at a time and maintain healthy mouth tissues, therefore they should be impressed with the necessity for periodical visits. A great majority need prophylactic treatment every four months, many every three months, some every two months and a few every month.

It should be constantly remembered that the prevention of dental disease depends largely upon the ability of patients to properly care for the toilet of the mouth, and that before their intelligent co-operation can be expected definite instructions must be given.—*Dental Items of Interest.*

